# Influence of titanium dioxide nanoparticles and/or cadmium chloride oral exposure on testicular morphology, oxidative stress, and apoptosis in rats: Ameliorative role of co-enzyme Q10

**DOI:** 10.1016/j.heliyon.2024.e24049

**Published:** 2024-01-03

**Authors:** Amany Behairy, Mohamed M.M. Hashem, Khaled Abo-EL-Sooud, Ahmed M. Soliman, Samar M. Mouneir, Abeer E. El-Metwally, Sameh H. Ismail, Bayan A. Hassan, Yasmina M. Abd-Elhakim

**Affiliations:** a Department of Physiology, Faculty of Veterinary Medicine, Zagazig University, Zagazig 44519, Egypt; b Department of Pharmacology, Faculty of Veterinary Medicine, Cairo University, Giza 12613, Egypt; c Pathology Department, Animal Reproduction Research Institute, Giza 3514805, Egypt; d Faculty of Nanotechnology for Postgraduate Studies, Cairo University, Sheikh Zayed Campus, 6th October City, Giza, 12588, Egypt; e Pharmacology Department, Faculty of Pharmacy, Future University, Cairo 11835, Egypt; f Department of Forensic Medicine and Toxicology, Faculty of Veterinary Medicine, Zagazig University, Zagazig 44519, Egypt

**Keywords:** Testis, Titanium dioxide nanoparticles, Cadmium chloride, Co-enzyme Q10, Caspase-3, Tumor necrotic factor α, **Oxidative stress**

## Abstract

**Background and objectives:**

Little is known about the implications of titanium dioxide nanoparticles (TiO_2_NPs) and cadmium chloride (Cd) co-exposure on the male reproductive system in mammals. As a result, this study researched the effects of oral TiO_2_NPs and/or Cd exposure on male reproduction and testicular functions. Additionally, a mitigation trial with co-enzyme Q10 (CoQ10) has also been conducted.

**Methods:**

In a 60-day experiment, seven experimental groups, each containing 10 male Sprague Dawley rats, were orally given distilled water (control), corn oil (vehicle control), CoQ10 (10 mg/kg b.wt), TiO_2_NPs (50 mg/kg b.wt), Cd (5 mg/kg b.wt), TiO_2_NPs + Cd, and TiO_2_NPs + Cd + CoQ10. Then, sperm quality, male sex hormones, oxidative stress indications, Ti and Cd testicular residues, testes and accessory gland architecture, and apoptotic and inflammatory markers in rat testes were assessed.

**Results:**

TiO_2_NPs and/or Cd exposure negatively impacted body weight, weight gain, testicular weights, semen quality, serum reproductive hormones, oxidative stress parameters, and Caspase-3 and tumor necrosis factor (TNF-α) immunoreactions. Histopathological changes were recorded in testicular, seminal vesicle, and prostatic tissues. Yet, co-administration of CoQ10 with TiO_2_NPs and Cd substantially mitigated these adverse consequences. The most notable aspect is that it effectively lowered testicular tissue Ti and Cd levels. It also improved oxidant status, hormonal profile, and sperm picture. CoQ10 minimized the testicular damage implied by histological examination. Furthermore, CoQ10 significantly diminished TiO_2_NPs and Cd-induced Caspase-3 and TNF-α immunoexpression in testicular tissue.

**Conclusion:**

As a result, CoQ10 could be utilized as a safe remedy to protect male reproductive physiology from TiO_2_NPs and Cd damage.

## 1 Introduction

Titanium dioxide nanoparticles (TiO_2_NPs) have become popular nanomaterials in a wide range of fields, including medicine, agriculture, bacteriostasis, wastewater management, personal care items, cosmetics, sunscreen, dental products, paint, and food [1]. TiO_2_NPs exposure can occur through inhalation, transdermal absorption, or ingestion, though oral intake is the most common, with a 75 kg adult human receiving 15–37.5 mg/kg/day through meals [2]. TiO_2_NPs entering the body may disrupt the oxidation balance and generate reactive oxygen species (ROS), giving rise to numerous detrimental outcomes such as reproductive toxicity [3], hepatotoxicity [4], and genotoxicity [5]. TiO_2_NPs exposure may promote inflammation and apoptosis, resulting in organ damage and failure [6].

TiO_2_NPs penetrate the testis via the blood-testis barrier (BTB) due to their tiny dimensions and then travel to the testicular microenvironment of Sertoli cells, sperm cells, and Leydig cells. They then destroy the typical architecture of the testis and inhibit testosterone production, resulting in a diminution in sperm quality; and causing a decrease in Leydig cells and sperm count, sperm motility, and other male fertility troubles [7]. In rats, TiO_2_NPs exposure has been reported to reduce spermatocytes, testosterone, and luteinizing hormone (LH) [8]. Moreover, in rats and mice, increased germ cell apoptosis and sperm abnormalities but declined sperm viability were recorded following TiO_2_NPs exposure [9]. Furthermore, disrupted spermatogenesis and steroid hormone gene expressions were evident in mice exposed to TiO_2_NPs [6].

Cadmium (Cd) is a common environmental and occupational metallic toxicant that harms humans' health [10,11]. Cd poisoning induces nephrotoxicity, osteoporosis, cardiovascular problems, testicular necrosis, renal failure, ocular toxicity, and neurological problems [12,13]. Additionally, Cd exposure causes severe edema, Leydig cell damage, decreased testosterone in the blood and testes, fewer germ cell junctions in the seminiferous tubules, a lack of integral membrane proteins at the Sertoli cell interface of the BTB, and a decrease in sperm motility and count [14]. Depletion of reduced glutathione (GSH) and protein-bound sulfhydryl groups are characteristic of Cd toxicity. These factors lead to increased ROS generation, including hydrogen peroxide, superoxide ions, and hydroxyl radicals, which may initiate lipid peroxidation [15]. Extensive necrosis of the seminiferous epithelium cells, hemorrhage, and interstitial tissue edema were triggered by Cd-induced inflammation in the testes, leading to male infertility [16].

Growing concerns have been directed toward using natural antioxidants to mitigate impaired fertility and testicular injury resulting from exposure to environmental pollutants [[17], [18], [19], [20]]. The co-enzyme Q10 (CoQ10) (2,3-dimethyl-6-ten-isoprene parabenzoquinone) is synthesized naturally in the body [21]. It is a fat-soluble biomolecule recognized in almost all microbes and mammals' mitochondria as an essential aspect of the electron transport chain and energy production [22]. CoQ10 is an antioxidant with anti-inflammatory and energy-boosting characteristics that can safeguard cells from apoptosis [23]. CoQ10 is extremely successful at preventing lipid, protein, and DNA oxidation. It also protects the cell from free radical oxidation [24]. CoQ10 exists naturally in seminal fluid and serves an important metabolic and antioxidant role in the seminal fluid as well as sperm motility, which necessitates a lot of energy [22,25]. CoQ10 preserves sperm lipids from peroxide destruction, encourages membrane stability, eliminates superoxide anions and peroxides, and performs vital in sperm development and maturing [26]. CoQ10 deficiency has been discovered to be linked with lowered sperm parameters [27].

Humans can be exposed to complex mixtures of environmental chemicals from various locations at the same time or in sequence. This study explored the single and combined impacts of TiO_2_NPs and Cd on reproductive health, reproductive hormonal status, testicular antioxidant status, and Caspase-3 and tumor necrosis factor (TNF-α) immunoreactions. The potential protective mechanism of CoQ10 against TiO_2_NPs and Cd-induced reprotoxic effects was also examined.

## 2 Materials and methods

### 2.1 Chemicals and reagents

Titanium dioxide nanoparticles (TiO_2_NPs) nanopowder (M.W = 79.87, 99.98 % purity, and average particle size of 6–19 nm) and cadmium chloride (CdCl_2,_ M.W = 183.32) were purchased from Alpha Chemika, Cairo, Egypt. Co-enzyme Q10 (CoQ10) was obtained from Mepaco-Medifood Co. (Cairo, Egypt). TiO_2_NPs suspension was freshly prepared in sterile distilled water. Also, Cd Cl_2_ was dissolved in distilled water. CoQ10 was diluted in corn oil (Arma Food Industries, 10th of Ramadan, Sharkia, Egypt). The rest of the chemicals and reagents used were of analytical quality and were bought from Sigma-Aldrich Co. in St. Louis, Missouri, USA.

### 2.2 Experimental design

Adult male Sprague Dawley rats (n = 70, average initial weight 163.86 ± 0.27, 12 weeks of age) were obtained from the National Research Center breeding section (Giza, Egypt). All rats were housed in well-ventilated, clean steel mesh cages and had a 12/12-h light/dark cycle with 50–60 % relative humidity and at 21–25 °C. A bedding made of wood shavings was used to keep the cages dry. Standard rodent pellets and tap water were freely available to the rats during the experiment. Before testing, rats were allowed to adapt to the laboratory environment for two weeks. Randomization of animals was performed, primarily based on animal weight, to ensure that initial animal weights do not exhibit any significant difference. The sample size was initially kept to 10 rats in each group, aiming to have a significance of 5 % and a power of 95 %. The sample size was calculated by the Resource equation method [28]. The experimental groups were as follows:

**G1: Control group:** received distilled water during the trial period.

**G2: Vehicle control group (Corn oil):** orally given 2 ml/kg b.wt corn oil.

**G3: Co-enzyme (CoQ10):** orally given 10 mg/kg b.wt CoQ10 dissolved in corn oil [29].

**G4: Titanium dioxide nanoparticles (TiO**_**2**_**NPs):** orally administered 50 mg/kg bwt TiO_2_NPs [30].

**G5: Cadmium chloride (Cd):** orally administered 5 mg/kg bwt Cd [31].

**G6: TiO**_**2**_**NPs + Cd:** co-administered TiO_2_NPs and Cd at the abovementioned doses.

**G7: TiO**_**2**_**NPs + Cd + CoQ10:** co-administered TiO_2_NPs, Cd, and CoQ10 at the above-mentioned doses.

All treatments were administered orally via orogastric gavage using a 16-gauge feeding needle once a day between 8 and 10 a.m. for 60 days. Rat spermatogenesis typically lasts between 56 and 60 days; thus, the experiment was designed to last the same time [32]. Each day, a new suspension of TiO_2_NPs was prepared, and the suspensions were sonicated in a distilled water bath using an ultrasonic cleaner (500 W, 42 kHz, 25 °C, FRQ-1010HT, Hangzhou, China) for 15 min and vortexed for 5 min before being used on the animals. Every week, the rats' weights were recorded.

### 2.3 Sample collection

After 60 days of dosing, the rats were weighed and anesthetized with an intraperitoneal administration of ketamine HCl (50 mg/kg, Sigma-Aldrich, St Louis, MO, USA) and xylazine (10 mg/kg, ELgomhoria CO., EGYPT). A fine, sterilized glass capillary tube was used to collect blood from each rat via the retro-orbital venous plexus [33]. Blood was withdrawn into a centrifuge tube without K_2_EDTA to obtain serum for hormone assessment. The rats were then decapitated, and their testes were quickly separated, freed of fat and connective tissue, and weighed. The absolute testicular weight was established using a sensitive weighing scale (Radwag, Model AS220/C/2, Clarkson Laboratory and Supply Inc., Chula Vista, CA, USA). The relative testicular weight was estimated using the formula: Relative testes weight = Testes weight/Body weight × 100 [34]. The testis samples were divided into three groups. The first was fixed in 10 % buffered formalin for histopathological evaluations and Caspase-3 and TNF-α immunohistochemical studies. A tissue homogenizer (Potter-Elvehjem, Thomas Scientific, Swedesboro, NJ, USA) homogenized the second group of testicular samples (0.5 g) in chilled potassium chloride. The resulting homogenate was centrifuged at 4 °C for 10 min at 3000 rpm. The supernatants were analyzed afterward to determine the levels of testicular enzymes and antioxidants. The last group was preserved at 4 °C till the Ti and Cd content was analyzed.

### 2.4 Semen analysis

The cauda epididymis was removed immediately after the rats were euthanized and cut into small pieces using sterile scissors, then mixed with 2 mL physiological saline preheated at 37 °C. Sperm concentration, motility, and abnormalities were assessed in the resultant suspension. The epididymal suspension was studied under a microscope at 40 magnification by placing a drop on a clean glass slide preheated to 37 °C and covering it with another clean glass slide that had also been preheated to 37 °C. Numerous microscopical fields were inspected within 2–4 min to assess about 200 sperm collected from the epididymis. The percentage of motile sperm cells was calculated by a subjective scoring system ranging from 0 to 100 % [35]. Sperm was counted using a hemocytometer chamber slide after being (1:4) diluted with normal physiological saline and treated with 4 drops of 40 % formalin to destroy the spermatozoa [36]. The sperm cell concentration in a semen sample of 1 mL was calculated by the following formula: *n ×* 10 *×* 5 *× dilution* factor *×* 1000. Given that *n* = the sperm number in a volume of 0.1 mm^3^ diluted semen [37]. The dilution factor of 25 was applied to avoid sperm cell overlapping and to promote sperm count for precise findings. The proportion of abnormal sperm was determined in eosin/nigrosine stained smears following the protocol of Filler [38]. On a glass slide, the formalin-mixed sperm solution (10 μL) was carefully mixed with a drop of nigrosine and 5 % eosin solution (15 μL). The smears were then made, air-dried, and studied under a microscope at a 400*×* magnification. A hundred spermatozoa per slide have been selected at random and analyzed for abnormalities in the three main regions (tail, neck/mid-piece, and head).

### 2.5 Male sexual hormones assessment

Serum levels of male sex hormones have been measured using commercially available rat enzyme-linked immunosorbent assay (ELISA) kits according to the supplied protocol. Cusabio Biotech Company provided rat testosterone ELISA kits with CSB-E05100r Catalogue no. (Range of detection (ng/mL): 0.13 to 25.6 and < 0.06 ng/mL sensitivity). While rat estradiol ELISA kits were obtained from Kamiya Biomedical Company (Seattle, WA, USA) with KT-14291 Catalogue no. (Range of detection (pg/mL): 12.35–1000 and < 4.38 pg/mL sensitivity). Also, rat FSH ELISA kits with KT-15332 Catalogue no. (Range of detection (ng/mL): 2.47–200 and 1.11 ng/mL sensitivity) and rat LH ELISA kits with KT-21064 Catalogue no. (Range of detection (ng/mL): 0.37–30 and 0.153 ng/mL sensitivity) were obtained from Kamiya Biomedical Company (Seattle, WA, USA).

### 2.6 Oxidative stress assessment

Biodiagnostic colorimetric bioassay kits (Dokki, Giza, Egypt) were employed for determining glutathione peroxidase (GPx) and superoxide dismutase (SOD) in line with **Paglia and Valentine** [39] and **Nishikimi et al.** [40]. MDA levels were identified by reacting it with thiobarbituric acid for 30 min at 95 °C in an acidic medium, producing a pink reactive product at 534 nm absorbance [41].

### 2.7 Analysis of Ti and Cd residues

The testicular samples were digested with 8 mL nitric acid and 1 mL of 30 % hydrogen peroxide in microwaves. An inductively coupled plasma-optical Emission Spectrometer (ICP-OES) model 5100 (Agilent, Santa Clara, CA, USA) was then used to determine the Ti and Cd contents. Each measurement series' intensity was checked against a blank and at least three standards from the Merck Company (Darmstadt, Germany). The instrument readings were validated, and the precision and accuracy of the metal measurements were ensured by comparing them to the National Institute of Standards and Technology (NIST) standard reference material for trace elements in a quality control sample.

### 2.8 Histopathological examination

The formalin-fixed testis, seminal vesicle, and prostate gland were dehydrated, xylene-cleared, and paraffin-blocked. The sections were subsequently stained with H & E following Suvarna et al. [42] protocol. Tissues were examined at various magnifications using a light microscope (Olympus, Tokyo, Japan). Additionally, spermatogenesis and testicular damage were evaluated in the stained sections using a numeric grading system, as previously described by Johnsen [43].

Using Johnsen's tubular biopsy score (JTBS), a semiquantitative analysis of spermatogenesis was performed in 20 seminiferous tubules from each testicular section at 40 × magnification. Testicular tubule sections in each group were graded on a scale of 1–10. In this grading, 1 = Lack of spermatogenesis and germ cells, 2 = absence of germ cells and presence of Sertoli cells only, 3 = only spermatogonia exist, 4 = few spermatocytes exist, 5 = Lack of spermatids and spermatozoa yet an abundance of spermatocytes, 6 = absence of spermatozoa and presence of only a few spermatids, 7 = absence of spermatozoa and presence of many spermatids, 8 = only a few spermatozoa are present, 9 = numerous spermatozoa and disordered tubules are found, 10 = exhibited full spermatogenesis and uniform structure. JTBS was calculated by dividing the scores by the total number of seminiferous tubules inspected.

### 2.9 Immunohistochemistry assessment

Paraffin sections were additionally generated for Caspase-3 and TNF-α immuno-staining, according to Ref. [44]. They were conducted using a digital imaging software Programme and examined under a light microscope to assess the intensity and distribution of positive cells. Positively stained cells were counted. Each group's mean ± SE was computed.

### 2.10 Statistical analysis

The Shapiro-Wilk test was used to assess data normality, whereas Levene's was employed to assess variance homogeneity. The data has been expressed as means ± S.E and was analyzed using One way ANOVA, followed by the post hoc Tukey test, which established significance when *p* < 0.05 was achieved. The statistical analyses were carried out using Prism 7.0 GraphPad (Graph-Pad, San Diego, CA, USA).

## 3 Results

### 3.1 Effects on body weight change and relative testicular weight

No mortalities were recorded throughout the experiment. Neither the TiO_2_NPs-exposed nor the Cd-exposed rats showed a significant change in body weight or body weight gain compared to the control group. While rats in the Cd + TiO_2_NPs group showed a significantly reduced body weight by 19 % (*P* = 0.005) and reduced weight gain by 60 % (*P* = 0.002) relative to the control group. Nonetheless, body weight (*P* = 0.005) and weight gain (*P* = 0.002) were significantly increased in the TiO_2_NPs + Cd + CoQ10-treated group than the Cd + TiO_2_NPs group to be 5 % and 7 %, respectively, compared to the control group **(**Table 1**)**.

**Table 1:** Effect of co-enzyme Q10 (CoQ10) oral dosing on body weight change, relative testicular weight, and sperm characteristics of male rats exposed to titanium dioxide nanoparticles (TiO_2_NPs) and/or cadmium chloride (Cd) for 60 days.

Estimated parameters	Experimental groups						
	Control	Vehicle control	CoQ10	TiO_2_NPs	Cd	TiO_2_NPs +Cd	TiO_2_NPs +Cd + CoQ10
Initial Body weight (g)	160.67 ±0.47	161.67 ±1.18	160.00 ±0.00	163.33 ±2.36	166.67 ±4.71	166.67 ±2.36	168.00 ±3.08
Final body weight (g)	250.67 ±10.02	251.00 ±1.78	245.00 ±14.71	273.33^#^ ±15.74	231.67 ±1.65	202.67* ±8.65	264.00^€^ ±13.95
Body weight change (g)	90.00 ±10.26	89.33 ±2.49	85.00 ±14.71	110.00^#^ ±13.50	65.00 ±6.36	36.00* ±6.53	96.00^€^ ±12.66
Testes weight (g)	1.85 ±0.05	1.77 ±0.08	1.75 ±0.06	2.10^#^ ±0.00	1.45* ±0.07	1.46* ±0.19	1.88^€^ ±0.07
Relative testicular weight (%)	0.74 ±0.02	0.70 ±0.03	0.72 ±0.04	0.78 ±0.04	0.63 ±0.03	0.72 ±0.09	0.73 ±0.06
Sperm count (x10^6^/mL)	69.00 ±1.15	67.00 ±1.73	72.00 ±3.79	41.00*^#^ ±1.53	50.00*^#^ ±1.53	29.67* ±1.20	57.33*^€^ ±1.45
Sperm motility (%)	90.33 ±1.45	90.00 ±0.58	93.33 ±1.67	61.00*^#^ ±1.15	43.33*^#^ ±0.88	32.67* ±0.88	67.33*^€^ ±1.76
Sperm abnormalities (%)	16.50 ±1.32	17.60 ±0.52	13.89 ±0.56	29.55*^#^ ±1.98	29.26*^#^ ±1.09	37.20* ±.77	22.60*^€^ ±1.09

Control group: orally received distilled water, vehicle control group: orally given 2 ml/kg b.wt corn oil, CoQ10: orally given 10 mg/kg b.wt CoQ10 dissolved in corn oil, TiO_2_NPs group: orally administered 50 mg/kg bwt TiO_2_NP, Cd group: orally administered 5 mg/kg bwt CdCl_2_, TiO_2_NPs + Cd: orally co-administered TiO_2_NPs and Cd at the abovementioned dose, and TiO_2_NPs + Cd + CoQ10: orally co-administered TiO_2_NPs, Cd, and CoQ10 at the above mentioned doses. *p < 0.05 vs control, #*p* < 0.05 vs TiO_2_NPs + Cd, and € *p* < 0.05 TiO_2_NPs + Cd vs TiO_2_NPs + Cd + CoQ10. The values shown are means ± SE. n = 10 rats/group.

The testicular weight was significantly (*P* = 0.001) decreased in both the Cd-only–exposed group (22 %) and the Cd + TiO_2_NPs co-exposed group (21 %) than the control group. Besides, a significant (*P* = 0.001) difference in the testicular weight was recorded in the TiO_2_NPs–only–exposed group and the Cd + TiO_2_NPs-co-exposed one. While the TiO_2_NPs + Cd + CoQ10-treated group showed a significant (*P* = 0.001) increase in the absolute testis weight than the Cd + TiO_2_NPs-exposed one **(**Table 1**)**. Yet, no significant change (*P* > 0.05) in the relative testicular weight was found among all experimental groups.

### 3.2 Changes in the spermiogram

**Fig. 1:**
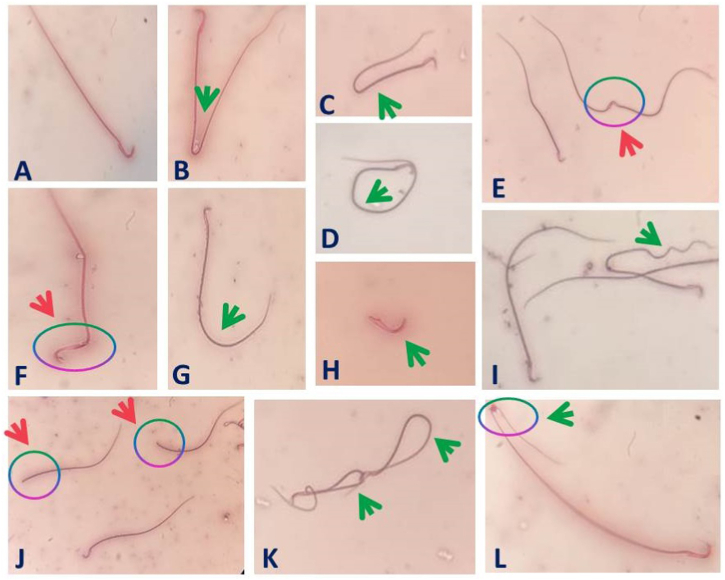
Representative photomicrographs of epididymal spermatozoa from male rats orally administered titanium dioxide nanoparticles and/or cadmium including abnormalities in sperm tail (Green arrow) and in sperm head (Pink arrow). (A) Normal sperm, (B), (C) Bent tail, (D) Looped tail, (E) Fused heads, (F) Bent head, (G) Curved tail, (H) Detached tail, (I) Curled tail, (J) Headless tail, (K) Coiled tail, (L) Bent tail with the protoplasmic droplet. (For interpretation of the references to color in this figure legend, the reader is referred to the Web version of this article.)

As shown in Table 1, the administration of both TiO_2_NPs, Cd, and their combination resulted in a significant (*P* < 0.001) decrease in the sperm motility percent by 32 %, 52 %, and 64 %, respectively, and in the sperm cell counts by 41 %, 28 %, and 57 %, respectively, compared to the control group. In contrast, the percentage of spermatozoa with abnormal morphology was significantly (*P* < 0.001) increased in TiO_2_NP (79 %), Cd (77 %), and TiO_2_NPs + Cd (126 %)-exposed rats compared to the control ones. The TiO_2_NPs + Cd co-exposed group showed a significant (*P* < 0.001) decrease in the sperm cell counts and motility but significantly (*P* < 0.001) higher sperms anomalies compared with the individual exposure (Table 1 and Fig. 1). Co-administration of CoQ10 with TiO_2_NPs and Cd significantly (*P* < 0.001) lessened the reductions in sperm motility (26 %) and sperm cells concentrations (17 %) but significantly (*P* < 0.001) lowered the percentage of abnormal spermatozoa (37 %) relative to the control group.

### 3.3 Effects on male sex hormones

Serum testosterone activity significantly (*P* = 0.003) reduced in TiO_2_NPs (36 %), Cd (43 %), and TiO_2_NPs + Cd (51 %) groups than the control group (Table 2). The most reduction in serum testosterone level was recorded in the TiO_2_NPs + Cd-co-exposed group. Nonetheless, there was a significant increase (*P* = 0.003) in serum testosterone concentration in the TiO_2_NPs + Cd + CoQ10 group to the TiO_2_NPs + Cd group and nearly similar to the control group (2 %).

**Table 2:** Effect of co-enzyme Q10 (CoQ10) oral dosing on serum levels of male hormones and testicular oxidative stress indicators and testicular tissue content of cadmium (Cd) and titanium dioxide nanoparticles (TiO_2_NPs) of male rats exposed to TiO_2_NPs and/or Cd for 60 days.

Estimated parameters	Experimental groups						
	Control	Vehicle control	CoQ10	TiO_2_NPs	Cd	TiO_2_NPs +Cd	TiO_2_NPs + Cd + CoQ10
Testosterone (ng/mL)	0.47 ±0.02	0.47 ±0.09	0.50 ±0.08	0.30* ±0.01	0.27* ±0.03	0.23* ±0.02	0.46^€^ ±0.03
FSH (ng/mL)	4.73 ±0.12	4.97 ±0.37	5.00 ±0.21	3.60*^#^ ±0.07	3.27* ±0.19	2.90* ±0.12	4.13*^€^ ±0.10
LH (ng/mL)	2.37 ±0.02	2.33 ±0.08	2.83* ±0.09	1.93*^#^ ±0.06	1.90*^#^ ±0.08	1.57* ±0.17	2.20^€^ ±0.04
Estradiol (pg/mL)	19.47 ±0.70	19.80 ±0.79	19.50 ±1.93	25.90^#^ ±0.41	29.43* ±3.24	35.17* ±3.45	22.40^€^ ±1.29
SOD (IU/g. protein)	44.67 ±1.49	47.70 ±0.55	54.91 ±1.19	15.40* ±1.49	15.71* ±2.02	13.25* ±1.69	41.95^€^ ±1.54
GPx (IU/g. protein)	95.34 ±2.27	96.74 ±0.37	135.66* ±3.95	47.83*^#^ ±4.01	39.06*^#^ ±0.11	19.52* ±0.05	75.33*^€^ ±5.90
MDA(nmol/g. protein)	124.95 ±1.61	126.17 ±1.12	108.75* ± 1.51	135.60*^#^ ±0.27	137.33*^#^ ±3.10	167.79* ±0.88	125.45^€^ ±1.07
Testicular Ti residues (ppm)	ND	ND	ND	0.56* ±0.04	ND	0.53* ±0.02	0.07*^€^ ±0.00
Testicular Cd residues (ppm)	ND	ND	ND	ND	0.18*^#^ ±0.01	0.40* ±0.01	0.09*^€^ ±0.00

LH: luteinizing hormone; FSH: follicle-stimulating hormone; SOD: superoxide dismutase; GPx: glutathione peroxidase; MDA: malondialdehyde; Ti: titanium. *p < 0.05 vs control, #*p* < 0.05 vs TiO_2_NPs + Cd, and € *p* < 0.05 TiO_2_NPs + Cd vs TiO_2_NPs + Cd + CoQ10. The values shown are means ± SE. n = 10 rats/group. Control group: orally received distilled water, vehicle control group: orally given 2 ml/kg b.wt corn oil, CoQ10: orally given 10 mg/kg b.wt CoQ10 dissolved in corn oil, TiO_2_NPs group: orally administered 50 mg/kg bwt TiO_2_NP, Cd group: orally administered 5 mg/kg bwt CdCl_2_, TiO_2_NPs + Cd: orally co-administered TiO_2_NPs and Cd at the abovementioned dose, and TiO_2_NPs + Cd + CoQ10: orally co-administered TiO_2_NPs, Cd, and CoQ10 at the above mentioned doses.

Serum FSH concentration significantly (*P* < 0.001) decreased in TiO_2_NPs (30 %), Cd (39 %), and TiO_2_NPs + Cd (49 %) groups than the control ones. The decrease in serum FSH hormone was more significant in the TiO_2_NPs and Cd group than in the individually TiO_2_NPs -exposed one. On the other hand, the TiO_2_NPs + Cd + CoQ10 group showed a significant (*P* < 0.001) increase in serum FSH than the TiO_2_NPs + Cd group and was nearly similar to the control group (16 %) (Table 2).

Serum LH concentration was significantly (*P* < 0.001) higher in the CoQ10 group by 19 % than the control value (Table 2). While, serum LH significantly (*P* < 0.001) decreased in the TiO_2_NPs (19 %), Cd (20 %), and TiO_2_NPs + Cd (34 %) groups than the control group. The maximum reduction in serum LH hormone was recorded in the TiO_2_NPs + Cd group. Nonetheless, there was a significant restoration (*P* < 0.001) in serum LH concentration in the TiO_2_NPs + Cd + CoQ10 groups than TiO_2_NPs + Cd group to a very near value to the control value (7 %) (Table 2).

The serum estradiol significantly (*P* < 0.001) increased in the Cd (51 %) and TiO_2_NPs + Cd (81 %) groups more than in the control group. The maximum increase in serum estradiol was observed in the TiO_2_NPs + Cd co-exposed group. There was a significant (*P* < 0.001) reduction in serum estradiol in the TiO_2_NPs + Cd + CoQ10 group than the TiO_2_NPs + Cd group.

### 3.4 Effects on testicular oxidative stress biomarkers

Individual administration of CoQ10 induced a significant (*P* < 0.001) increase in the testicular GPx (42 %) activity but a significant (*P* < 0.001) reduction (13 %) in testicular MDA than the control group (Table 2). On the other hand, testicular SOD activity significantly (*P* < 0.001) reduced in the TiO_2_NPs (66 %), Cd (65 %), and TiO_2_NPs + Cd (70 %) groups than the control one. Yet, testicular SOD activity significantly (*P* < 0.001) reestablished (6 %) in the TiO_2_NPs + Cd + CoQ10 group relative to the control group. In comparison to the control group, testicular GPx activity significantly (*P* < 0.001) reduced by 50 % in the TiO_2_NPs group, 59 % in the Cd group, and 80 % in the TiO_2_NPs + Cd group (Table 2). Nevertheless, GPx testicular activity significantly (*P* < 0.001) restored (21 %) in the TiO_2_NPs + Cd + CoQ10 group relative to the control group.

Testicular MDA significantly (*P* < 0.001) increased in the TiO_2_NPs (9 %), Cd (10 %), and TiO_2_NPs + Cd (34 %) groups compared to the control group (Table 2). The rats co-administered TiO_2_NPs and Cd together showed a more significant (*P* < 0.001) decline in GPx concentrations with a more significant (p < 0.05) increase in MDA levels than rats administered each individually. On the contrary, MDA concentration significantly (*P* < 0.001) decreased in the TiO_2_NPs + Cd + CoQ10 group, similar to the control value (0.40 %).

### 3.5 Effects on testicular concentrations of Ti and Cd

Rats exposed to TiO_2_NPs alone or in combination with Cd revealed a significant (*P* < 0.001) increase in the testicular Ti residues compared with the normal control group (Table 2). A non-significant (*P* > 0.05) difference was recorded in testicular Ti residues between the TiO_2_NPs or TiO_2_NPs + Cd group. Yet, the TiO_2_NPs + Cd + CoQ10 group presented a significant (*P* < 0.001) decrease in testicular Ti residues than the TiO_2_NPs + Cd group.

Concerning testicular Cd residues, rats exposed to Cd alone or combined with TiO_2_NPs for 60 consecutive days exhibited a significant (*P* < 0.001) increase in Cd residues compared with the normal control group, as presented in Table 2. The Cd and TiO_2_NPs co-exposed group exhibited greater testicular Cd residues. The co-administration of CoQ10 for Cd and TiO_2_NPs intoxicated male rats for 60 consecutive days displayed a significant (*P* < 0.001) decrease in testicular Cd residues to rats administered Cd + TiO_2_NPs **(**Table 2**).**

### 3.6 Effects on testicular and accessory glands histopathology

#### 3.6.1 Testicular tissue

**Fig. 2:**
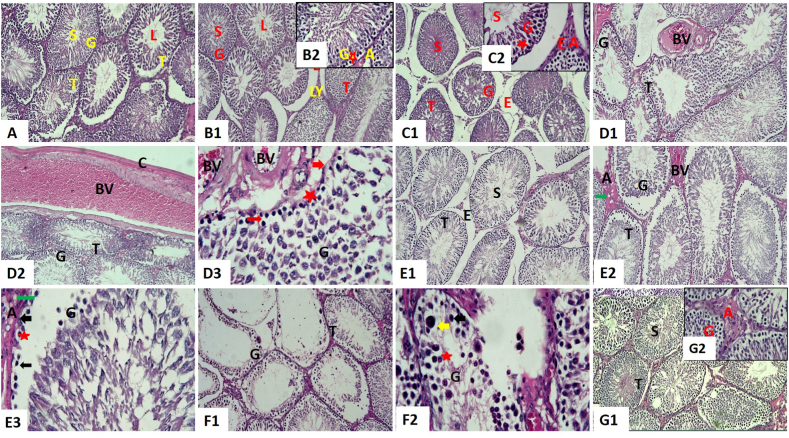
Representative histopathological photomicrographs of hematoxylin-stained cross-sections of rat testes. Control group **(A,** X100**)**, co-enzyme Q10-treated group **(B1,** X100 and **B2**, X400**),** corn oil-treated group **(C1,** X100 and **C2**, X400**),** titanium dioxide nanoparticles-exposed group **(D1 and D2,** X100 and **D3**, X400**),** cadmium-exposed group **(E1 and E2,** X100 and **E3**, X400**)**, titanium dioxide nanoparticles and cadmium co-exposed group **(F1,** X100 and **F2**, X400**), and** titanium dioxide nanoparticles and cadmium co-exposed group treated with co-enzyme Q10 **(G1,** X100 and **G2**, X400**).** Acidophilic material (A), blood vessels (BV), cellular vacuolations (star), darkly stained pyknotic nuclei (thin arrow), epithelial vacuolations (star), germinal epithelium (G), giant spermatids (yellow arrow), interstitial edema (E), Leydig cells (LY), mild interstitial edema (E), seminiferous tubules (T), spermatozoa (S), thickened basement membrane (thick arrow), thickened capsule (C), tubular lumen (L), and vacuolation of Leydig cells (green arrow). (For interpretation of the references to color in this figure legend, the reader is referred to the Web version of this article.)

Examination of tissues of the control group showed normal structure. They consisted of seminiferous tubules lined by numerous germinal epithelium layers and spermatozoa in the tubular lumen. Both Sertoli and spermatogenic cells were found in the germinal epithelium. There was a clear progression of spermatogenic cells from spermatogonia to primary spermatocytes to spermatids to mature spermatozoa, all of which were regularly organized from the basal compartment to the lumen. The basement membrane of all seminiferous tubules was quite thin. Elongated pyramidal Sertoli cells were located, separating spermatogenic cells as supporting cells. Also, Leydig cells and blood vessels were present in the interstitial tissue (Fig. 2A).

The testicular tissue architecture of both groups’ administrated CoQ10 (Fig. 2 B1 and B2) and corn oil (Fig. 2C1 and C2) were similar to the examined control tissues. Vaculations were present in some tubules, and spermatozoa filled the lumen. Deposition of acidophilic material in interstitial tissue was observed. Some of the interstitial blood vessels were congested. Edema was prominent in the group given corn oil.

Compared with the control group, the testis of rats treated with TiO_2_NPs (Fig. 2 D1, D2, and D3) or Cd (Fig. 2 E1, E2, and E3) revealed pathological changes at different severity. Seminiferous tubules were atrophied with unequal outlines and had sloughed germinal epithelium from the basement membrane. Also, several sections had disordered, darkly pigmented, pyknotic nuclei and vacuolation in their germinal epithelium. A markedly thickened basement membrane was obvious. Sertoli cells appeared necrosed. There were vacuolations in Leydig cells. Severe congestion and thickening in the wall with sub-capsular and interstitial blood vessel vacuolations were seen, especially in the group treated with TiO_2_NPs. However, there was interstitial edema. Deposition of acidophilic material in interstitium was observed. Thickening in the capsule was also seen. In addition, Cd induced a few multinucleated giant cells. The lesions were most severe in rats exposed to TiO_2_NPs.

Regarding the TiO_2_NPs and Cd co-exposed group, testicular tissue displayed obvious pathological changes (Fig. 2 F1 and F2) compared to the control group. The germinal epithelium disorganization was pronounced with spermatogenesis vacuolations and cell interruption. Few or no spermatogenic cells were present, and atrophic tubules contained only Sertoli cells, indicating loss of the germinal epithelium. Besides, giant spermatids, both uninucleated and multinucleated, were conspicuous, and cellular debris accumulated in the tubules. A markedly thickened basement membrane, congestion, and interstitial edema were more prominent. Vacuolated acidophilic material in the interstitium was observed. Prominent thickening in the capsule was seen. As shown in Fig. 2 G1 and G2, CoQ10 treatment reversed the histopathological changes in rats co-exposed to TiO_2_NPs and Cd, where spermatogenesis was active, and their testes appeared normal. Few tubules showed spermatogenic disorganization. Vacuolated deposition of acidophilic material in interstitium was still present.

As mentioned in Table 3, there was no statistically significant (*P* > 0.05) change in JTBS between the control group and the group treated with CoQ10 and corn oil. In comparison, TiO_2_NPs and/or Cd-exposed groups had a significant (*P* < 0.001) decrease in JTBS than the control group. Yet, the TiO_2_NPs + Cd9+ CoQ10 and control groups did not differ significantly (*P* > 0.05) statistically in JTBS.

**Table 3:** Effect of co-enzyme 10 exposure (CoQ10) on testicular Johnsen's tubular biopsy score (JTBS) and immunoexpression of Caspase-3 and tumor necrotic factor alpha (TNF-α) of male rats exposed to titanium dioxide nanoparticles (TiO_2_NPs) and/or cadmium chloride (Cd) for 60 days.

	Control	Vehicle control	CoQ10	TiO_2_NPs	Cd	TiO_2_NPs +Cd	TiO_2_NPs + Cd + CoQ10
JTBS	9.40 ±0.11	9.50 ±0.11	9.55 ±0.11	5.85*^#^ ±0.17	6.05*^#^ ±0.22	2.80* ±0.24	9.05^€^ ±0.16
Caspase-3	19.67 ±2.78	17.00 ±1.87	22.00 ±2.94	37.67* ±1.31	33.67*^#^ ±2.25	42.00* ±2.86	16.00^€^ ±2.12
TNF-α	11.33 ±1.31	11.67 ±1.03	9.33 ±1.31	25.00*^#^ ±1.41	21.67*^#^ ±1.55	29.00* ±0.41	15.67*^€^ ±1.25

*p < 0.05 vs control, #*p* < 0.05 vs TiO_2_NPs + Cd, and € *p* < 0.05 TiO_2_NPs + Cd vs TiO_2_NPs + Cd + CoQ10. The values shown are means ± SE. n = 10 rats/group. Control group: orally received distilled water, vehicle control group: orally given 2 ml/kg b.wt corn oil, CoQ10: orally given 10 mg/kg b.wt CoQ10 dissolved in corn oil, TiO_2_NPs group: orally administered 50 mg/kg bwt TiO_2_NP, Cd group: orally administered 5 mg/kg bwt CdCl_2_, TiO_2_NPs + Cd: orally co-administered TiO_2_NPs and Cd at the abovementioned dose, and TiO_2_NPs + Cd + CoQ10: orally co-administered TiO_2_NPs, Cd, and CoQ10 at the above mentioned doses.

#### 3.6.2 Prostate glands

**Fig. 3:**
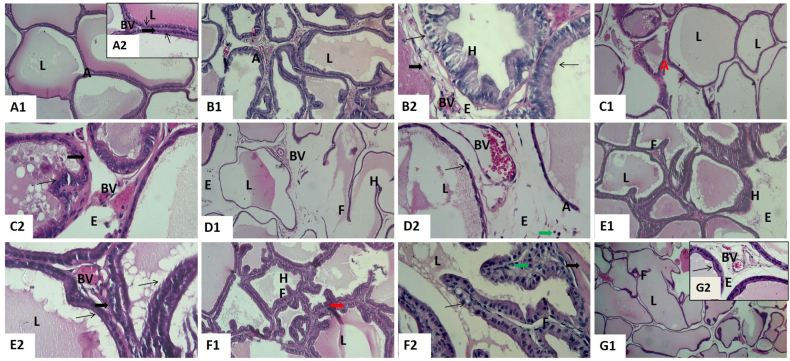
Representative histopathological photomicrographs of hematoxylin-stained cross-sections of rat prostate glands. Control group **(A1,** X100 and **A2**, X400**)**, co-enzyme Q10-treated group **(B1,** X100 and **B2**, X400**),** corn oil-treated group **(C1,** X100 and **C2**, X400**),** titanium dioxide nanoparticles-exposed group **(D1,** X100 and **D2**, X400**),** cadmium-exposed group **(E1,** X100 and **E2**, X400**)**, titanium dioxide nanoparticles and cadmium co-exposed group **(F1,** X100 and **F2**, X400**), and** titanium dioxide nanoparticles and cadmium co-exposed group treated with co-enzyme Q10 **(G1,** X100 and **G2**, X400**).** Acini (A), acinar hyperplasia (H), acinar lumen (L), blood vessels (BV), edema (E), epithelial hyperplasia (H), fibromuscular stroma (thick arrow), inflammatory cells (green arrow), interstitial edema (E), papillary folds (F), prostatic acinar hyperplasia (H), simple columnar epithelium (thin arrow), and thick fibro-muscular stroma (thick arrow). (For interpretation of the references to color in this figure legend, the reader is referred to the Web version of this article.)

The prostate gland of control (Fig. 3 A1 and A2), CoQ10 (Fig. 3 B1 and B2), and corn oil (Fig. 3C1 and C2) groups showed normal glandular tissue. The latter consisted of aggregations of numerous tubuloalveolar acini of different sizes and shapes surrounded by a thick external capsule. Their acini were lined with cuboidal epithelium or simple columnar with basal nuclei, and their lumen enclosed prostatic secretions. Blood vessels were located in the secretory tubules incorporated in the fibromuscular stroma. In addition, there was mild epithelial hyperplasia and blood vessel congestion in the prostate gland of CoQ10 and corn oil groups.

On the other side, the prostate gland of rats administered either TiO_2_NPs (Fig. 3 D1 and D2) or Cd (Fig. 3 E1 and E2) or both (Fig. 3 F1 and F2) for 60 days exhibited many histopathological alterations than the control group. The prostatic acini were formed of the vacuolated flattened epithelium; some had pronounced papillary projections towards the lumen and decreasing acinar size. Destruction of some acini was seen. Scanty vacuolated secretions were also found in their lumens. Most of the interstitial blood vessels were congested. There was interstitial edema. An obvious thick fibro-muscular layer and monocellular inflammatory cells around the acini were seen. The prostate of rats co-exposed to TiO_2_NPs and Cd accompanied with CoQ10 revealed normal acini and mild epithelial hyperplasia. Inside the lumen, vacuolated secretion was observed. Also, there was congestion and minimal edema (Fig. 3 G1 and G2).

#### 3.6.3 Seminal vesicles

**Fig. 4:**
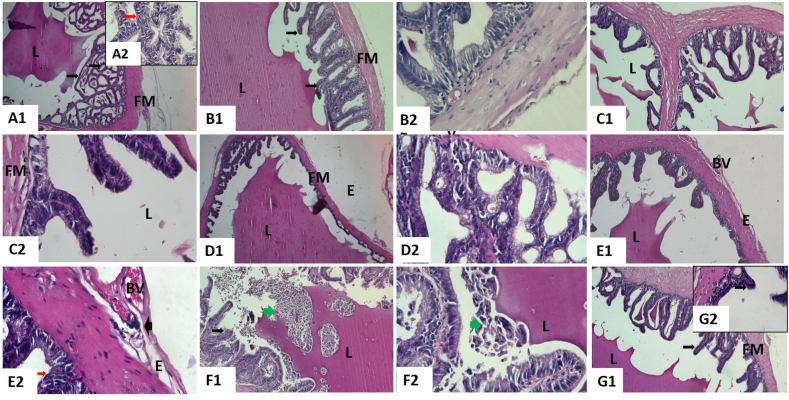
Representative histopathological photomicrographs of hematoxylin-stained cross-sections of rat seminal vesicles. Control group **(A1,** X40 and **A2**, X400**)**, co-enzyme Q10-treated group **(B1,** X100 and **B2**, X400**),** corn oil-treated group **(C1,** X100 and **C2**, X400**),** titanium dioxide nanoparticles-exposed group **(D1,** X100 and **D2**, X400**),** cadmium-exposed group **(E1,** X100 and **E2**, X400**)**, titanium dioxide nanoparticles and cadmium co-exposed group **(F1,** X100 and **F2**, X400**), and** titanium dioxide nanoparticles and cadmium co-exposed group treated with co-enzyme Q10 **(G1,** X100 and **G2**, X400**)**. Blood vessels (BV), branched mucosal folds (arrows), edema (E), epithelial lining (red arrows), fibro-muscular layer (FM), lumen (L), monocellular inflammatory cells (arrowheads), and mucosal folds (arrows). (For interpretation of the references to color in this figure legend, the reader is referred to the Web version of this article.)

The examination of the seminal vesicles of control (Fig. 4 A1 and A2), CoQ10 (Fig. 4 B1 and B2), and corn oil (Fig. 4C1 and C2) groups of the adult male rats revealed that the normal histological structure. It resembled branching, convoluted folds of mucosa encircled by smooth muscle. Their folds are lined with pseudostratified columnar epithelium with greatly vesicular nuclei and foamy cytoplasm, and the lumen is filled with secretions. The gland is protected by a strong fibro-muscular capsule, through which the trabeculae emerge. Blood vessels were slightly congested in the seminal vesicles of CoQ10 and corn oil groups.

On the other hand, TiO_2_NPs and/or Cd-exposed groups revealed pathological alterations compared with the control (Fig. 4 D1, D2, E1, E2, F1, and F2). The number and height of mucosal folds were noticeably increased, forming very coiled hyperplasic folds. A conspicuous vacuolated epithelium was found. Destruction of some folds was seen. Moreover, the fibro-muscular layer displayed thickness, edema, congested blood vessels, vacuolations, and monocellular inflammatory cell infiltrations. Very few secretions were observed within the acini lumen. Significant edema under the capsule, vasculitis, and congestion of the capsular blood vessels were also evident. Obviously, these alterations were seen in that group treated with TiO_2_NPs and Cd. Seminal vesicles of rats treated with TiO_2_NPs and Cd with CoQ10 revealed normal structure, mild epithelial hyperplasia, and papillary projections. Also, there was mild edema and congestion (Fig. 4 G1 and G2)

### 3.7 Immunohistochemistry of Caspase-3 and TNF-α

**Fig. 5:**
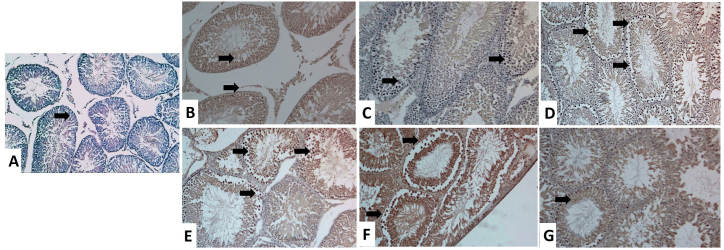
Representative histopathological photomicrographs of testicular tissue sections stained for Caspase-3 immunoexpression (X 100). **(A)** Control group. **(B)** Co-enzyme Q10-treated group. **(C)** Corn oil-treated group. **(D)** Titanium dioxide nanoparticles-exposed group. (E) Cadmium-exposed group. **(F)** Titanium dioxide nanoparticles and cadmium co-exposed group. **(G)** Titanium dioxide nanoparticles and cadmium co-exposed group treated with co-enzyme Q10. Caspase-3 positive reactions in the cytoplasm of spermatogenic cells and Leydig cells are represented by a brown color (arrows). (For interpretation of the references to color in this figure legend, the reader is referred to the Web version of this article.)

**Fig. 6:**
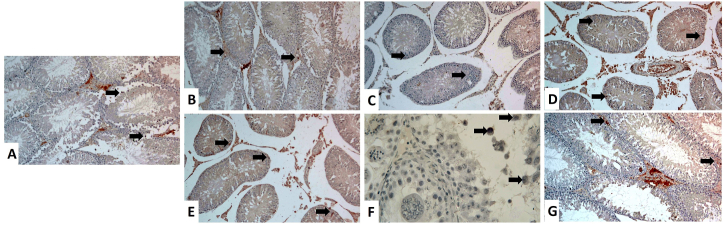
Representative histopathological photomicrographs of testicular tissue sections stained for TNF-α immunoexpression (X 100). **(A)** Control group. **(B)** Co-enzyme Q10-treated group. **(C)** Corn oil-treated group. **(D)** Titanium dioxide nanoparticles-exposed group. (E) Cadmium-exposed group. **(F)** Titanium dioxide nanoparticles and cadmium co-exposed group. **(G)** Titanium dioxide nanoparticles and cadmium co-exposed group treated with co-enzyme Q10. TNF-α positive reactions in the cytoplasm of spermatogenic cells and Leydig cells are represented by brown color (arrows). (For interpretation of the references to color in this figure legend, the reader is referred to the Web version of this article.)

As demonstrated in Fig. 5, Fig. 6 and Table 3, a significantly (*P* < 0.001) increased Caspase-3 protein expression was evident in TiO_2_NPs (Fig. 5D), Cd (Fig. 5E), and TiO_2_NPs + Cd (Fig. 5F) groups than the control (Fig. 5A), CoQ10 (Fig. 5B), and corn oil (Fig. 5C) groups. The positive apoptotic cells (dark brown) were observed in Leydig, Sertoli, and germinal epithelium. In addition, spermatids showed a weak Caspase-3 positive reactivity. On the other side, the Caspase-3 immunoreactivity reaction in the TiO_2_NPs + Cd + CoQ10 group (Fig. 5G) was more or less similar to the control group.

In addition, compared to the control (Fig. 6A), CoQ10 (Fig. 6B), and corn oil (Fig. 6C) groups, there was a significant (*P* < 0.001) elevation of TNF-α expression in testes of TiO_2_NPs (Fig. 6D), Cd (Fig. 6E), and TiO_2_NPs + Cd (Fig. 6F) co-exposed rats. In contrast, groups treated with CoQ10 resulted in significant (*P* < 0.001) decreases in testicular TNF-α expression compared to the control group. TNF-α immunoreactivity reaction was similar to the control group in the TiO_2_NPs + Cd + CoQ10 (Fig. 6G) group.

## 4 Discussion

The current study findings showed that rats exposed to Cd alone or in combination with TiO_2_NPs had significantly lower body weight change and reduced testicular weight. The Cd-induced necrotic and degenerative changes could explain the loss of body weight and testicular weight via derangement in some testicular constructions, such as seminiferous tubules and Leydig cells, that typically account for 70 %–80 % of the testicular mass [45]. Moreover, the Cd-induced lipid peroxidation and oxidative damage could contribute to reproductive organ atrophy [46]. Yet, herein, rats given TiO_2_NPs exhibited a significant increase in body weight gain and testicular weight. This could be explained by the recent mechanistic studies that reported that TiO_2_NPs oral exposure induced colonic mucus layer disturbance and obesity-related microbiota dysbiosis [47]. On the contrary, CoQ10 co-administration with TiO_2_NPs and Cd nearly recovered changes in body weight gain and testicular weight to normal control due to modified testicular morphology enhanced by CoQ10.

Both TiO_2_NPs and/or Cd exposure had a detrimental impact on sperm characteristics in this study. In this respect, Wang et al. [48] reported that Cd causes impairment in the physiological function of sperm-specific cation channel that serves as the primary source of intracellular Ca^2+^, as well as a sperm-specific potassium channel, which both are important for regulating sperm physiology and subsequently lowered viability and motility of sperm. Moreover, the histopathological findings revealed that testicular sections of the Cd-exposed group showed an abundance of multinucleated giant cells. Additional DNA replication of primary spermatocytes that do not undergo meiosis causes giant cell formation [49]. Additionally, the reduction in sperm quality caused by Cd may be due to either a malfunction of the H_2_O_2_ removal system, which leads to hindering steroidogenesis in the Leydig cells due to an excess of H_2_O_2_, or to membrane harm or macromolecular deterioration caused by ROS [50]. Furthermore, TiO_2_NPs might block ATP synthase, leading to lowered ATP content and disrupted motor capabilities in the sperm [51]. TiO_2_NPs prompt androgen deficits, creating estrogen imbalance, testicular disorders, and suppressing spermatogenesis [52,53]. On the contrary, when 50 mg/kg bwt TiO_2_NPs and 5 mg/kg bwt Cd were co-administered with 10 mg CoQ10 for 60 days, there was an adequate enhancement of sperm concentration, motility, progressive motility, and morphology compared to the TiO_2_NPs + Cd groups. CoQ10 safeguards sperm lipids from peroxide damage, boosts membrane integrity, eradicates superoxide anion and peroxides, and assists in sperm maturation and development [54]. The antioxidant effect of CoQ10 on cells and sperm function contributes to producing energy for the spermatozoa, boosting human sperm motility [26].

Here, serum FSH, LH, and testosterone concentrations were significantly lower in TiO_2_NPs, Cd, and TiO_2_NPs + Cd exposed rats, with a substantial rise in estradiol concentration. TiO_2_NPs' negative effects on Leydig cells, the key synthesis site for testosterone hormone, may be to blame for a decline in testosterone manufacture. TiO_2_NPs interrupt the oxidative balance in the testes of Sprague Dawley rats, which may be the basis of testosterone synthesis restriction [52]. Additionally, Gao et al. [6] stated that TiO_2_NPs exposure reduced testosterone production by lowering FSH and LH generated by the pituitary gland, contributing to Sertoli cell injury. A substantial decline in serum testosterone levels could be a result of Cd-induced reduced production and accessibility of cholesterol for steroidogenesis or as a result of steroidogenic enzyme downregulation, which plays a vital role in steroidogenesis [49]. In addition, Cd, as a potent xenoestrogen, influences anterior pituitary secretion via a feedback loop or by directly promoting cellular death in anterior pituitary cells [50]. Cd stays in the hypothalamus and pituitary gland, exciting oxidative stress and adversely influencing these organs' hormonal secretions [55]. The current study's inverse changes in testosterone and estradiol levels could be ascribed to a rise in aromatase enzyme levels which participates in a critical step in the conversion of androgens to estrogens, which can result in lower testosterone levels and higher estradiol levels in the blood [56]. The observed swings in serum testosterone and estradiol levels may also be ascribed to fluctuations in testicular TNF-α concentration. TNF-α has been shown to boost the production of cytochrome P450 aromatase in germ cells. On the contrary, CoQ10 treatment significantly raised serum hormonal concentrations compared to the TiO_2_NPs + Cd group, implying that CoQ10 could play an integral part in the hormone-regulated manufacturing of testosterone. The improvement in testosterone levels after CoQ10 treatment could be attributed to CoQ10's bioenergetic properties, as it regulates the activity and output of testicular Leydig cells by influencing the energy production and electron transport chain in their mitochondrial membranes [57]. Second, CoQ10 may protect testosterone-producing cells from oxidative stress by scavenging ROS and inhibiting membrane lipid peroxidation [58]. A human study found that CoQ10 treatment significantly raised the FSH and LH in infertile men, suggesting that CoQ10 may be associated with hormone-regulated testosterone synthesis [59].

Both TiO_2_NPs and Cd, particularly in the co-exposed group, could cross the BTB and deposit in the seminiferous cord, where they were translocated to Sertoli cells and/or the nucleus, causing severe pathological changes that support the current hypothesis. TiO_2_NPs-induced spermatogenic deterioration might contribute to the buildup of free radical products in testicular cells as reported by Morgan et al. [60]. The latter also investigated the histopathological changes provoked by TiO_2_NPs in the seminal vesicle and prostate and discovered that NPs caused desquamation, congestion, and hyperplasia of the prostate's epithelial lining and seminal vesicle congestion. Cd-prompted sloughing and disorganization with incomplete spermatocytes' arrest [61]. In contrast, CoQ10 co-supplementation with TiO_2_NPs and Cd reestablished reproductive tissue architecture and optimized testicular damage biomarkers.

In this study, TiO_2_NPs and/or Cd exposure produced a substantial drop in SOD and GPx testicular activities and a substantial rise in MDA testicular content than the control group. TiO_2_NPs, as ROS producers, caused spermatogenic damage [9]. NPs fluctuate electron transfer by raising the NADP+/NADPH ratio and disrupting mitochondrial function, generating intracellular ROS [62]. Comparably, TiO_2_NPs significantly reduced catalase, SOD, GPX, glutathione reductase, and glutathione S-transferase activities but elevated ROS production in male mice testicular tissue [63]. Cd testicular toxicity can be exacerbated via various mechanisms, including glutathione exhaustion, Cd binding to sulfhydryl groups on cell membrane proteins, lowering antioxidant enzyme activities, ROS generation, and increased tissue lipid peroxidation [50]. On the contrary, the testicular activities of the antioxidants enzyme (SOD and GPx) and MDA level improved when CoQ10 was given concurrently to TiO_2_NPs and Cd-exposed animals. CoQ10 is a fat-soluble antioxidant that may penetrate directly into the polyunsaturated lipid chain of the plasma membrane, safeguarding the spermatozoa's plasma membrane [26]. It can also remove free radicals that harm DNA, proteins, and lipids [64]. Increasing sperm concentrations of CoQ10 and its reduced form, ubiquinol, is the primary function of CoQ10 in the testis [27]. Ubiquinol is a potent fat-soluble antioxidant that can eradicate peroxyl radicals generated via lipid peroxidation and replenish vitamins E and C [65].

Herein, Cd accumulation was revealed by the higher amount of Cd in the testis of rats co-exposed to Cd and TiO_2_NPs. The higher Cd concentration in the Cd + TiO_2_NPs co-exposed group confirmed TiO_2_NPs' potentiating effect on Cd-induced testicular injury. The increased accumulation of Ti and/or Cd could be attributed to daily metal delivery, which ensures continuous absorption throughout the study period. Disruption of the BTB, a major target of Cd toxicity in the testis, and its underlying mechanisms of action are highlighted in studies of Cd-induced testicular injury [66]. TiO_2_NPs prompt oxidative stress, which alters the content of BTB-associated and actin-regulatory proteins, impacting tight junction and cytoskeleton structure [67]. The previous authors verified that TiO_2_NPs could widen the BTB gaps, allowing small TiO_2_NPs to cross and affect spermatogenesis. On the other hand, CoQ10 co-administration with TiO_2_NPs and Cd significantly reduced TiO_2_NPs and/or Cd residue in testicular tissues, which could be attributed to CoQ10's antioxidant properties. CoQ10 aids in detoxification and membrane stabilization, as well as protecting the testes from electromagnetic fields, oxidative stress, and ischemia/reperfusion impairment [68].

Apoptosis is frequently linked to oxidative stress. In the present study, increased cleaved Caspase-3 activity in the TiO_2_NPs, Cd, and TiO_2_NPs + Cd testis indicates a greater rate of germ cell apoptosis and, as a result, lesser spermatogenesis. Similarly, in peripheral blood lymphocytes, TiO_2_NPs elevated ROS and activated pro-apoptotic proteins such as Caspase 9 and 3 [69]. Cd exposure causes oxidative stress, which stimulates the mitochondrial pathway of apoptosis. ROS production and accumulation disrupt Ca^+2^ channel function, affecting mitochondrial membrane permeability and cytochrome *c* release into the cytoplasm. In the presence of adenosine triphosphate, cytochrome *c* can activate apoptotic protease activating factor-1, leading to Caspase-9 activation. Caspase-9 proteolysis of Caspase-3 zymogen triggers Caspase-3 activation and DNA fragmentation [70]. When CoQ10 was combined with TiO_2_NPs and Cd, the immunoactivity of Caspase-3 was reduced. The ability of CoQ10 to suppress DNA breakage and mitochondrial depolarization while increasing ATP levels accounts for its antiapoptotic properties. Furthermore, CoQ10 inhibits apoptosis-inducing substances from translocating to the nucleus by suppressing mitochondrial complex I activity [71].

TNF-α immunoexpression significantly increased in rats given TiO_2_NPs, Cd, or TiO_2_NPs + Cd than in control animals. This increase may be connected to the activation of nuclear factor kappa B (NF-κB), which causes inflammatory processes that harm testicular tissue. Cd-induced the NF-κB signaling pathway by dissociating from its inhibitory IκBα (inhibitory kappa B). This signaling increases the production of proinflammatory cytokines like TNF-α [72]. Yet, rats co-administered CoQ10 with both TiO_2_NPs and Cd showed a reduction in TNF-α immunoexpression compared to the TiO_2_NPs, Cd, and TiO_2_NPs + Cd treatment groups. Our findings could be attributed to CoQ10's anti-inflammatory properties. In this respect, Sohet et al. [73] demonstrated that CoQ10 reduced hepatic mRNA expression of TNF-α. Likewise, Schmelzer et al. [74] suggested that CoQ10 might decrease proinflammatory cytokine generation by inhibiting NF-κB gene expression.

## 5 Conclusion

The present study findings displayed that hormonal dysregulation, oxidative stress, and apoptosis are involved in testicular toxicity caused by TiO_2_NPs and/or Cd bioaccumulation, particularly at their concurrent exposure. Additionally, CoQ10 (10 mg/kg b.wt) reduced the negative effects of Cd and TiO_2_NPs on male fertility, altered sexual hormone levels, body weight, and testicular weights, sperm count, viability, and motility in male rats. Moreover, CoQ10 effectively reduced TiO_2_NPs and Cd-induced testicular damage and dysfunction in rats. Furthermore, CoQ10's antioxidant, antiapoptotic, and anti-inflammatory properties are thought to be responsible for its reproductive protection. Therefore, we propose that CoQ10 administration may be used as a preventative measure for persons at high risk of TiO_2_NPs and Cd exposure, such as those employed in the industrial sector.

## Data Availability statement

All data generated or analyzed during this study are included in this published article.

## Funding

This research was funded by Cairo University in a project entitled "Assessment of the risk hazards of co-exposure to nanomaterials and environmental contaminants with mitigation strategies using natural products" (Cairo university projects-12-2021).

## Ethical approval

The animal studies described later were carried out in accordance with the National Institutes of Health's general criteria for the care and use of laboratory animals in scientific investigations, and they were approved by Cairo University's research committee on animal ethics (Approval no. VET CU 2009 2022 462). Furthermore, the research was conducted in accordance with the ARRIVE criteria [75].

## Consent to participate

Not applicable.

## Consent to publish

Not applicable.

## CRediT authorship contribution statement

**Amany Behairy:** Conceptualization, Data curation, Methodology, Visualization, Writing - original draft. **Mohamed M.M. Hashem:** Conceptualization, Funding acquisition, Investigation, Methodology, Project administration, Resources, Writing - review & editing. **Khaled Abo-EL-Sooud:** Conceptualization, Funding acquisition, Investigation, Methodology, Project administration, Resources, Writing - review & editing. **Ahmed M. Soliman:** Conceptualization, Investigation, Methodology, Writing - review & editing. **Samar M. Mouneir:** Conceptualization, Investigation, Methodology, Writing - review & editing. **Abeer E. El-Metwally:** Conceptualization, Data curation, Investigation, Methodology, Visualization, Writing - review & editing. **Sameh H. Ismail:** Data curation, Methodology, Software, Validation, Visualization, Writing - review & editing. **Bayan A. Hassan:** Conceptualization, Data curation, Resources, Validation, Visualization, Writing - review & editing. **Yasmina M. Abd-Elhakim:** Conceptualization, Data curation, Formal analysis, Funding acquisition, Investigation, Methodology, Writing - review & editing.

## Declaration of competing interest

The authors declare that they have no known competing financial interests or personal relationships that could have appeared to influence the work reported in this paper.
